# Association between timing of speech and language therapy initiation and outcomes among post-extubation dysphagia patients: a multicenter retrospective cohort study

**DOI:** 10.1186/s13054-022-03974-6

**Published:** 2022-04-08

**Authors:** Takashi Hongo, Ryohei Yamamoto, Keibun Liu, Takahiko Yaguchi, Hisashi Dote, Ryusuke Saito, Tomoyuki Masuyama, Kosuke Nakatsuka, Shinichi Watanabe, Takahiro Kanaya, Tomoya Yamaguchi, Tetsuya Yumoto, Hiromichi Naito, Atsunori Nakao

**Affiliations:** 1 Department of Emergency, Okayama Saiseikai General Hospital, 2-25 Kokutaityo, Okayama Kita-ku, Okayama 700-8511 Japan; 2 Department of Emergency, Critical Care, and Disaster Medicine, Okayama University Graduate School of Medicine, Dentistry, and Pharmaceutical Sciences, 2-5-1 Shikata-cho, Okayama Kita-ku, Okayama 700-8558 Japan; 3 Department of Healthcare Epidemiology, School of Public Health, Graduate School of Medicine, Kyoto University, Yoshida-honmachi, Kyoto Sakyo-ku, Kyoto 606-8501 Japan; 4 Critical Care Research Group, Faculty of Medicine, University of Queensland, The Prince Charles Hospital, 627 Rode Rd, Chermside, Brisbane, QLD 4032 Australia; 5 Department of Intensive Care Medicine, Kameda Medical Center, 929 Higashicho, Kamogawa, Chiba 296-0041 Japan; 6 Department of Emergency and Critical Care Medicine, Seirei Hamamatsu General Hospital, 2-12-12 Sumiyoshi, Hamamatsu Naka-ku, Shizuoka 430-8558 Japan; 7 Department of Emergency, Misato Kenwa Hospital, 4-494-1 Takano, Misato, Saitama 341-0035 Japan; 8 Department of Anesthesiology, Okayama Rosai Hospital, 1-10-25 Chikkomidorimachi, Okayama Minami-ku, Okayama 702-8055 Japan; 9 Department of Rehabilitation, Nagoya Medical Center, NHO, 4-1-1 Sannomaru, , Nagoya Naka-ku, Aichi 461-0001 Japan; 10 Department of Rehabilitation, Hokkaido Medical Center, NHO, 7-1-1 Yamanote5jo, Sapporo Nishi-ku, Hokkaido 063-0005 Japan; 11 Division of Critical Care Medicine, Nara Prefecture General Medical Center, 2-897-5 Shichijonishimachi, Nara, Nara 630-8581 Japan

**Keywords:** Post-extubation dysphagia, Speech and language therapy, Intensive care, Dysphagia, Aspiration pneumonia

## Abstract

**Background:**

Post-extubation dysphagia (PED) is recognized as a common complication in the intensive care unit (ICU). Speech and language therapy (SLT) can potentially help improve PED; however, the impact of the timing of SLT initiation on persistent PED has not been well investigated. This study aimed to examine the timing of SLT initiation and its effect on patient outcomes after extubation in the ICU.

**Methods:**

We conducted this multicenter, retrospective, cohort study, collecting data from eight ICUs in Japan. Patients aged ≥ 20 years with orotracheal intubation and mechanical ventilation for longer than 48 h, and those who received SLT due to PED, defined as patients with modified water swallowing test scores of 3 or lower, were included. The primary outcome was dysphagia at hospital discharge, defined as functional oral intake scale score < 5 or death after extubation. Secondary outcomes included dysphagia or death at the seventh, 14th, or 28th day after extubation, aspiration pneumonia, and in-hospital mortality. Associations between the timing of SLT initiation and outcomes were determined using multivariable logistic regression.

**Results:**

A total of 272 patients were included. Of them, 82 (30.1%) patients exhibited dysphagia or death at hospital discharge, and their time spans from extubation to SLT initiation were 1.0 days. The primary outcome revealed that every day of delay in SLT initiation post-extubation was associated with dysphagia or death at hospital discharge (adjusted odds ratio (AOR), 1.09; 95% CI, 1.02–1.18). Similarly, secondary outcomes showed associations between this per day delay in SLT initiation and dysphagia or death at the seventh day (AOR, 1.28; 95% CI, 1.05–1.55), 14th day (AOR, 1.34; 95% CI, 1.13–1.58), or 28th day (AOR, 1.21; 95% CI, 1.07–1.36) after extubation and occurrence of aspiration pneumonia (AOR, 1.09; 95% CI, 1.02–1.17), while per day delay in post-extubation SLT initiation did not affect in-hospital mortality (AOR, 1.04; 95% CI, 0.97–1.12).

**Conclusions:**

Delayed initiation of SLT in PED patients was associated with persistent dysphagia or death. Early initiation of SLT may prevent this complication post-extubation. A randomized controlled study is needed to validate these results.

**Supplementary Information:**

The online version contains supplementary material available at 10.1186/s13054-022-03974-6.

## Background

Approximately 13–20 million critically ill patients are intubated in the intensive care unit (ICU) every year [1]. Post-extubation dysphagia (PED), the inability to swallow food or liquid following extubation, is a major complication among patients in the ICU [2, 3]. An early study estimated its incidence as up to 62% of critically ill patients with recent prolonged intubation [3]. Importantly, a study showed that 87% of patients with PED failed to recover from dysphagia at ICU discharge, and 64% of those patients had persistent swallowing disorders at hospital discharge [4]. Even at six months, 23% of PED patients still experienced dysphagia [5]. Additionally, PED can negatively impact patient outcomes, leading to longer hospital length of stay, aspiration pneumonia, re-intubation, need to place feeding tubes, and higher mortality rate [4, 6–8]. These findings emphasize the importance of multidisciplinary efforts in the early recognition and management of PED; however, to date, limited evidence exists to treat this pathology in the ICU.

A recent meta-analysis showed that swallowing treatments had a significant effect on reducing the risk of developing pneumonia in the critical care setting [9]. However, most data were derived from stroke patients treated with electrical stimulation. Speech and language pathologists (SLPs), experts in the treatment of communication disorders or swallowing impairments, primarily evaluate and manage patients with dysphagia in the ICU [10, 11]. Although SLPs are supposed to play a critical role in treating this complication, the optimal timing of interventions for PED patients by SLPs is still unclear. Early oral trials may cause a transient decrease in oxygen saturation, while delay in interventions may result in less effectiveness and worse functional outcomes [9]. Some patients even recover without any specific treatment. These clinical concerns are causing a conflict between recovery of swallowing function and the risk of aspiration in the absence of any established evidence for this issue. More importantly, potential barriers to the care of PED patients might stem from the fact that this complication is still under-recognized and less of a focus among ICU staff [2, 12].

Therefore, we hypothesized that the timing of swallowing therapy by SLPs would affect the outcomes of PED patients. Accordingly, this study aimed to investigate distributions of the timing of swallowing therapy initiation by SLPs and evaluate its impact on the swallowing function of PED patients.

## Methods

The study was approved by the Ethics Committee of Okayama Saiseikai General Hospital (Approval number: 210506), and it conforms to the provisions of the Declaration of Helsinki. Patient consent was waived for all participants enrolled in this study because of the retrospective study design. This study was conducted following the Strengthening the Reporting of Observational Studies in Epidemiology (STROBE) guidelines.

### Study design and setting

This multicenter, retrospective, cohort study was performed using data from the electronic medical records of patients admitted to the ICUs of five tertiary and three community hospitals in Japan from January 2017 to December 2020**.** The five tertiary hospitals included Kameda Medical Center in Chiba, Seirei Hamamatsu General Hospital in Shizuoka, Nagoya Medical Center in Aichi, Hokkaido Medical Center in Hokkaido, and Nara Prefecture General Medical Center in Nara. The three community hospitals included Okayama Saiseikai General Hospital in Okayama, Misato Kenwa Hospital in Saitama, and Okayama Rosai Hospital in Okayama. The detailed characteristics of the institutions are shown in Additional file 1.

### Study population

All patients aged ≥ 20 years were eligible if they required orotracheal intubation and mechanical ventilation for longer than 48 h and were treated for dysphagia by SLPs because of a lower modified water swallowing test (MWST) score following extubation (described below). To focus on the patients with PED, those admitted to the ICU due to cerebrovascular disease, those who required tracheostomy, and those with preexisting dysphagia were excluded. Patients with treatment restrictions, ICU readmissions, and incomplete primary outcome data were also excluded. Patients’ dysphagia was determined to be preexisting if they had a history of aspiration pneumonia, frequent episodes of cough upon liquid consumption or food sticking in the throat, or required dietary modification before hospital admission. Nurses routinely screened post-extubation swallowing function using a bedside swallowing screening. Briefly, the MWST was used to screen for dysphagia and score swallowing on a scale of 1–5 by having the patient swallow 3 mL of water. We adopted the MWST because it has been widely used in Japan with its sensitivity and specificity of 0.70 and 0.88, respectively, and demonstrated significant association with post-extubation pneumonia in cardiovascular surgery patient [13–15]. The MWST scores were defined as follows: 1. no drinking with choking and/or respiratory distress; 2. drinking and respiratory distress; 3. drinking and choking and/or hoarseness without respiratory distress; 4. drinking without choking and respiratory distress; and 5. drinking without choking and respiratory distress, plus able to perform two repetitive dry swallows within 30 s [15, 16]. Patients with MWST scores of 3 or lower, which were associated with a greater risk of aspiration pneumonia, were subjected to swallowing therapy by SLPs [15, 16]. The timing of speech and language therapy (SLT) initiation was determined based upon the intensivist’s or attending physician’s discretion.

### SLT and exposures

In this study, SLT was defined as at least one unit (equal to 20 min) of rehabilitation conducted by SLPs during hospitalization [17]. The sessions included swallowing and/or compensatory rehabilitation strategies, both of which are common approaches to treating dysphagia [18]. Swallowing rehabilitation involved traditional swallowing exercises to restore the strength and coordination of the tongue, pharyngeal, and laryngeal muscles, as well as respiratory muscle strength training. Swallowing compensatory strategies refer to bolus modification (i.e., texture, volume, and consistency), environmental arrangement, or maintaining proper posture of the head, neck, and body before swallowing [9]. During hospitalization, SLPs were primarily responsible for modifying the diet, changing maneuvers, or making decisions to restrict oral intake when necessary. For those patients whose swallowing function was severely impaired, direct therapy involving swallowing texture-modified foods or liquids was suspended to avoid aspiration. In all the institutions, actual steps or orders were determined solely by clinical evaluations in the ICU and flexible endoscopic evaluation of swallowing (FEES) or videofluoroscopic swallowing study (VFSS) was used accordingly after ICU discharge. As an adjunctive tool for swallowing evaluation, repetitive saliva swallowing test was also used to help determine the appropriate intervention or food modification [19]. The main exposure was the days from extubation to SLT initiation. These data were obtained from electronic medical records.

### Data collection

We collected the following data from patients’ medical records: demographics (age, gender, and body mass index (BMI); comorbidities based on Charlson Comorbidity Index scores, including preexisting dementia and cerebrovascular disease; ICU admission source (outpatient, emergency department, general ward, transfer from another hospital); ICU admission category (cardiovascular, pulmonary, gastrointestinal, trauma, or other); ICU admission type (medical or surgery); presence of sepsis or septic shock based on Sepsis-3 criteria; Acute Physiologic and Chronic Health Evaluation (APACHE) II scores; Sequential Organ Failure Assessment (SOFA) scores at ICU admission; the presence of delirium and SOFA score on the day of extubation; MWST score following extubation; duration of mechanical ventilation; use of vasopressor, intra-aortic balloon pump (IABP), extracorporeal membrane oxygenation (ECMO), or renal replacement therapy (RRT); data on enteral nutrition (EN) and parenteral nutrition (PN) and SLT (timing, frequency, swallowing and/or compensatory rehabilitation); duration of oral intake after extubation; and outcomes including functional oral intake scale (FOIS) score (see below) at hospital discharge and on the seventh, 14th, and 28th day after extubation, aspiration pneumonia after extubation, in-hospital mortality, and length of ICU and hospital stay.

### Outcomes

The primary outcome was dysphagia or death at hospital discharge. This composite outcome was chosen based upon the previous studies to minimize selection bias by encompassing all patients who underwent SLT [20, 21]. Dysphagia was defined as FOIS score < 5, which represents a severely impaired oral intake [22, 23]. The FOIS, a validated and reliable scale for measuring dysphagia, was examined by the treating SLPs. Scores range from 1 (nothing by mouth) to 7 (total oral diet with no restrictions), with higher scores indicating better swallowing function [22]. Secondary outcomes included dysphagia or death at the seventh, 14th, and 28th day after extubation, aspiration pneumonia following extubation, and in-hospital mortality.

### Statistical analyses

Continuous variables are presented as median and interquartile ranges (IQRs), while categorical variables are presented as numbers and percentages. To assess the impact of timing of SLT initiation on patient outcomes, multivariable logistic regression was performed, adjusting for covariates including institutions, age, ICU admission type, comorbidities including preexisting dementia and cerebrovascular disease, duration of mechanical ventilation, delirium on the day of extubation, SOFA score on the day of extubation, EN, and PN. These variables were essentially selected based on the results of previous studies and our clinical interests [24]. The results were expressed as odds ratios (ORs) and 95% confidence intervals (CIs).

We then performed five sensitivity analyses. (1) We used alternative definitions of dysphagia as FOIS score < 6 (total oral diet with multiple consistencies, without special preparation but with specific food limitations) according to the previous study [25]. (2) We excluded patients who received gastrointestinal surgery to avoid possible confounding by oral intake restrictions. (3) We excluded those who died after extubation and received SLT because the association between dysphagia and fatality is assumed to be bidirectional: dysphagia can result from fatality. (4) To address other potential confounders of dysphagia, we selected different covariates to assess the robustness of our findings; we conducted additional multivariable logistic regression analyses to assess the association of SLT initiation timing and patient outcomes. In Model 2, gender, age, BMI, SOFA score at ICU admission, sepsis, and MWST score were added. Model 3 incorporated the duration of mechanical ventilation, vasopressor, ECMO, IABP, RRT, EN, and PN. (5) We conducted an analysis dividing the patients into three groups based on the time interval from extubation to SLT initiation to reassess the outcomes.

A *p*-value of < 0.05 was considered statistically significant. Statistical analysis was performed using Stata version 17 (StataCorp LP, College Station, TX).

## Results

**Fig. 1:**
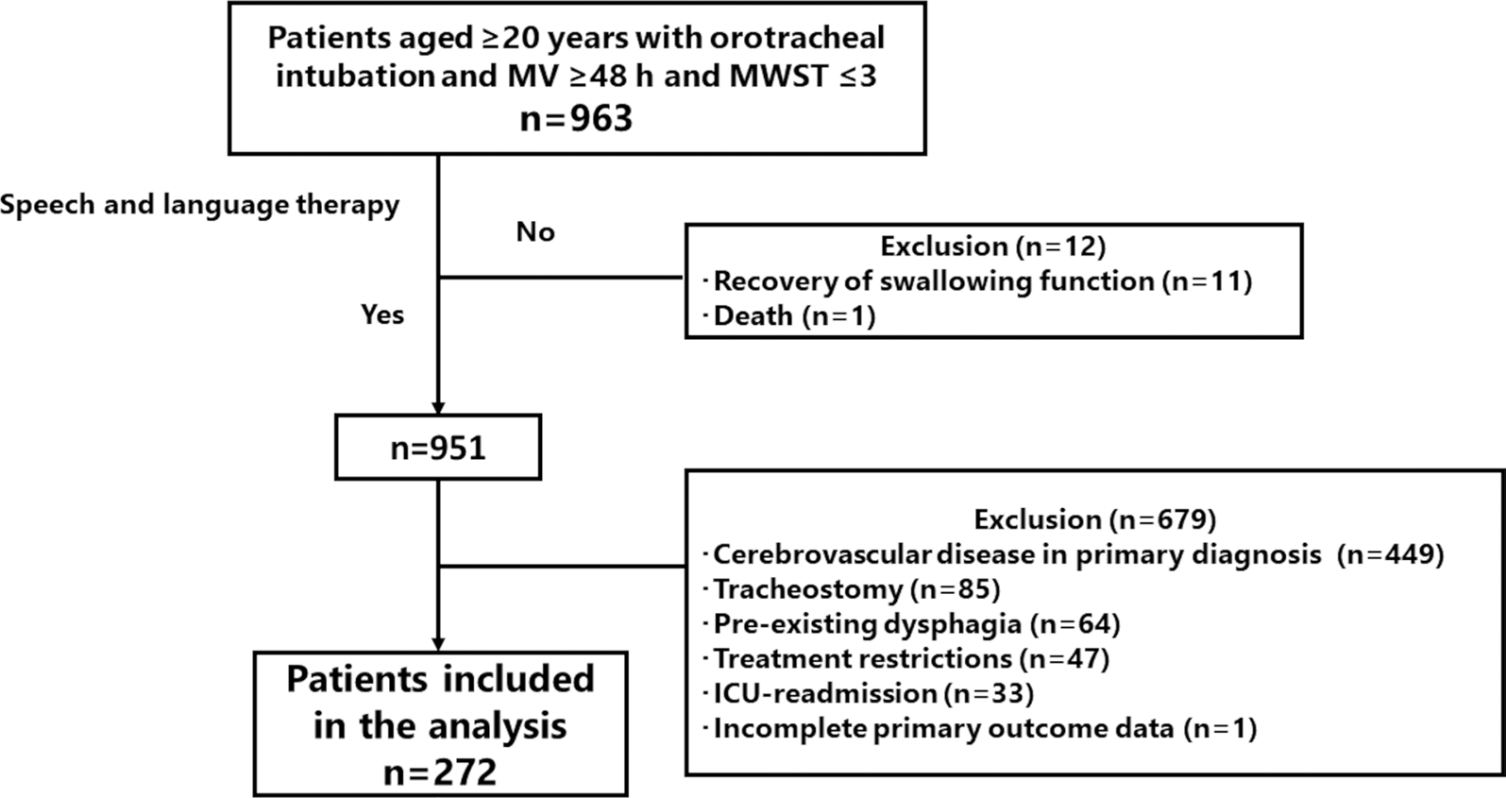
Flowchart of our study. MV: mechanical ventilation, MWST: modified water swallowing test, ICU: intensive care unit

During the four-year period, 963 adult patients (age ≥ 20 years) with MWST scores ≤ 3 following extubation from mechanical ventilation for more than 48 h were identified. After excluding cases (n = 691), 272 patients were included in our study cohort (Fig. 1).

**Fig. 2:**
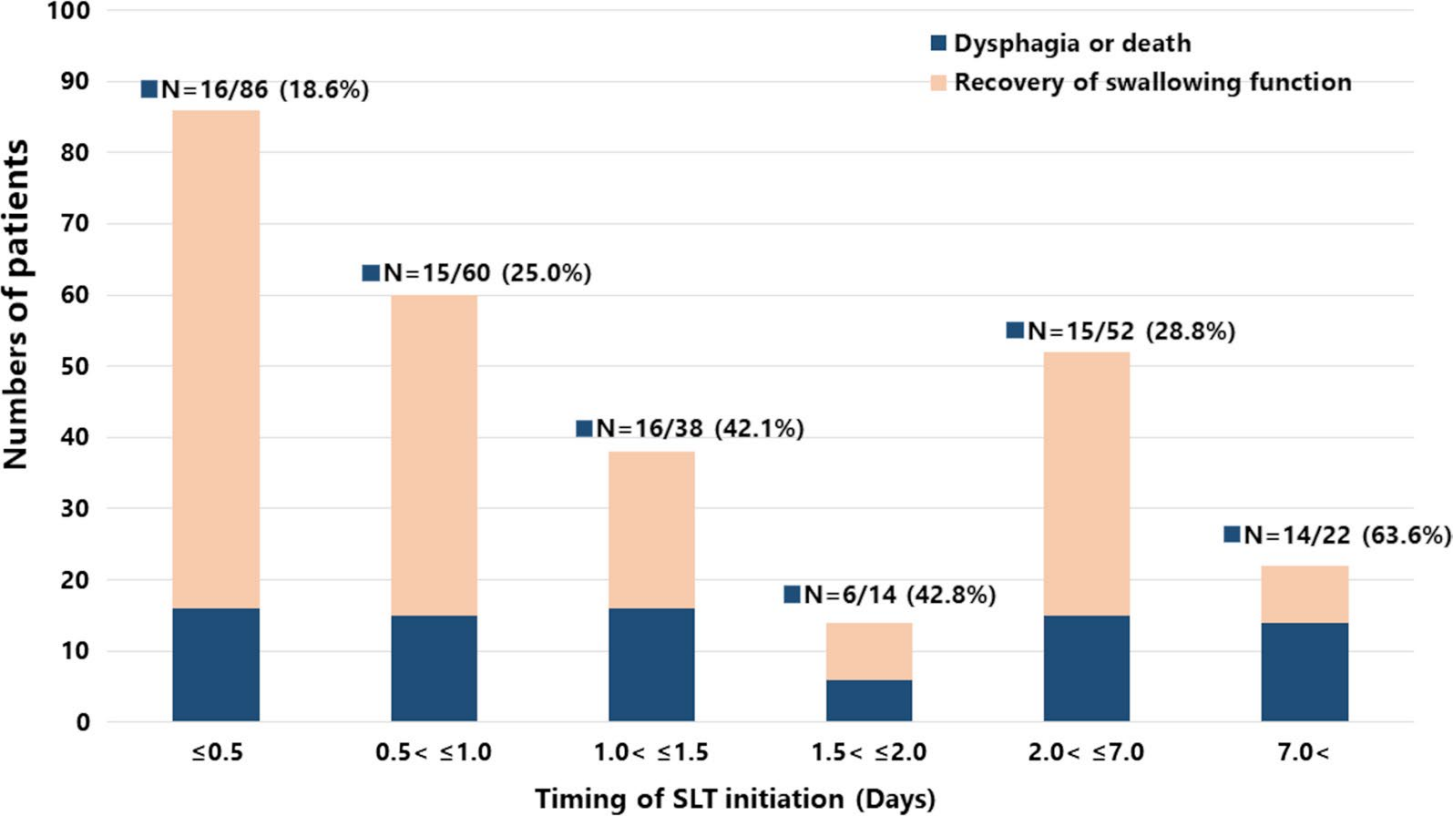
Distribution of the number of patients with dysphagia or death at hospital discharge based on the time interval from extubation to SLT initiation. SLT: speech and language therapy

**Fig. 3:**
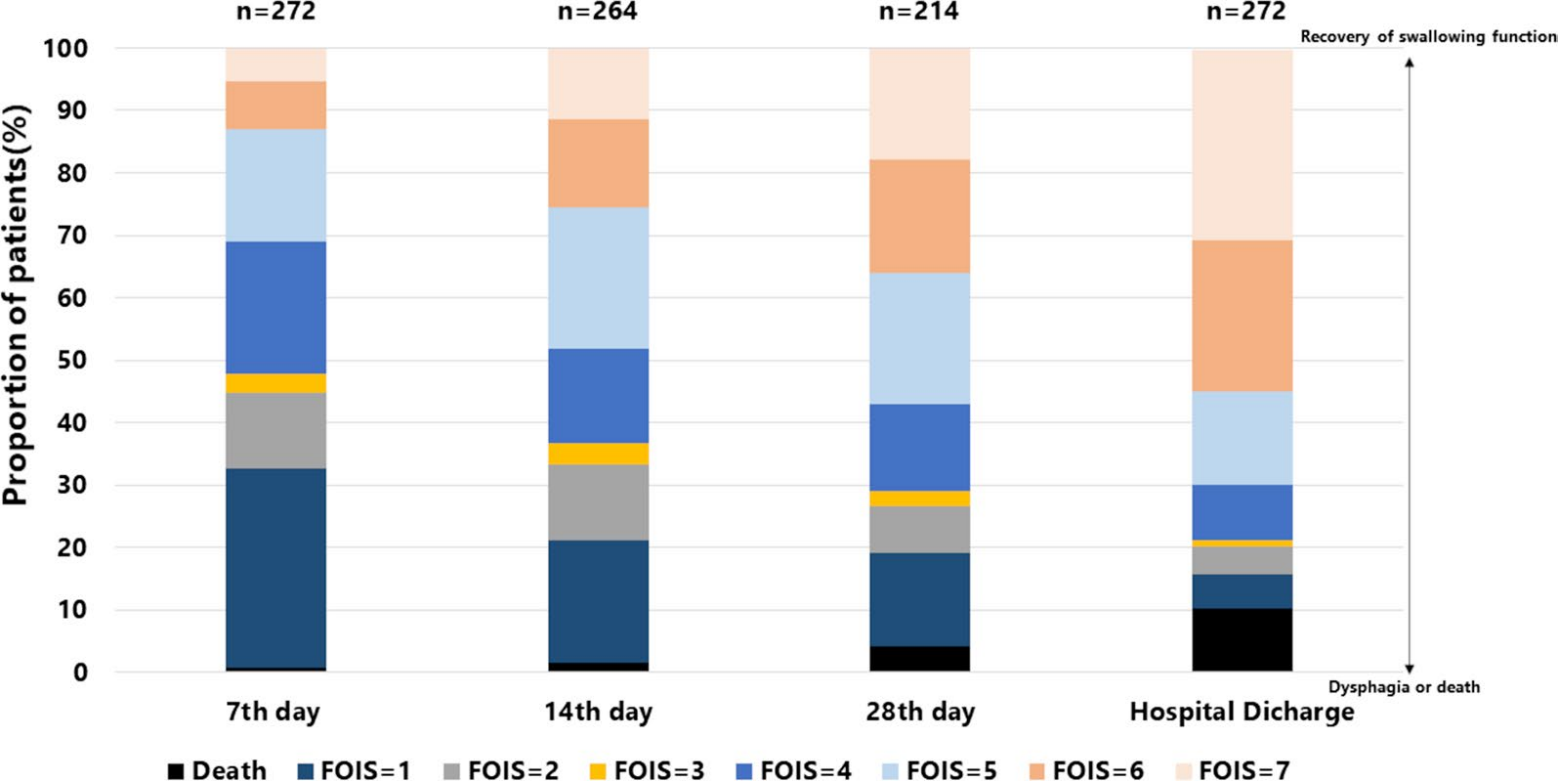
Distribution of FOIS scores and their trajectories over time. Early discharge from the hospital did not allow us to follow up eight and 58 patients on the 14th and 28th days after extubation, respectively. FOIS: function oral intake scale

The demographics and clinical characteristics of the participants are listed in Table 1. The median age of the participants was 74 years old (IQR 65–81); 188 (69.1%) were men and 84 (30.9%) were women. The prevalence of preexisting dementia and cerebrovascular disease was 14.3% (39/272) and 12.1% (33/272), respectively. The numbers of ICU admissions related to cardiovascular, respiratory, and gastrointestinal disease were 34 (12.6%), 88 (32.7%), and 56 (21.9%), respectively. Of the 272 patients included in the study, more than half had sepsis or septic shock; 114 (42.1%) underwent surgery. The patients were severely ill as shown by a median SOFA score of 8 (IQR 6–10) and a median APACHE II score of 23 (IQR 17–29) at ICU admission. The median duration of mechanical ventilation was 5.3 days (IQR 3.3–7.5). The swallowing assessment and characteristics of SLP intervention are shown in Table 2. The median MWST score on the first day of swallow screening was 3 (IQR 2–3). The median intervals from extubation to SLT initiation and extubation to oral intake were one day (IQR 0.3–2.2) and four days (IQR 2–9), respectively. Figure 2 describes the proportion of patients presenting with dysphagia or death at discharge based on the number of days from extubation to SLT initiation. Although there was a slight increase over time in the number of patients who died after initiating SLT, Fig. 3 demonstrates a gradual improvement of swallowing function: median FOIS scores on the seventh, 14th, and 28th days after extubation and at hospital discharge were 4 (IQR 1–5), 4 (IQR 2–6), 5 (IQR 3–6), and 6 (IQR 5–7), respectively. The prevalence of dysphagia or death at hospital discharge was 30.1% (82/272) (Table 3). Table 3 demonstrates the association of SLT initiation timing and outcomes. After adjusting for covariates (i.e., institutions, age, ICU admission type, comorbidities including preexisting dementia and cerebrovascular disease, duration of mechanical ventilation, delirium on the day of extubation, SOFA score on the day of extubation, EN, and PN), the primary outcome revealed that every day of delay in SLT initiation after extubation was associated with dysphagia or death at hospital discharge (adjusted odds ratio (AOR), 1.09; 95% CI, 1.02–1.18). Similar results were obtained at different time points: on the seventh day (AOR, 1.28; 95% CI, 1.05–1.55), 14th day (AOR, 1.34; 95% CI, 1.13–1.58), and 28th day (AOR, 1.21; 95% CI, 1.07–1.36) after extubation. In addition, the timing of SLT initiation (per day delay) was associated with the occurrence of aspiration pneumonia (AOR, 1.09; 95% CI, 1.02–1.17), but it was not associated with in-hospital mortality (AOR, 1.04; 95% CI, 0.97–1.12).

**Table 1:** Patient characteristics of the whole cohort

Variables	All (*n* = 272)
*Clinical information*	
Male, gender, n (%)	188 (69.1)
Age–median [IQR], years	74 [65–81]
BMI–median [IQR], kg/m^2^	21.0 [18.4–23.9]
Charlson comorbidity index–median [IQR]	2 [1–3]
Dementia, *n* (%)	39 (14.3)
Cerebrovascular disease, *n* (%)	33 (12.1)
*ICU admission from*	
Outpatients, *n* (%)	19 (7.0)
Emergency department, *n* (%)	173 (63.6)
General ward, *n* (%)	65 (23.9)
Transfer from another hospital, *n* (%)	15 (5.5)
*ICU admission type*^a^	
Medical, *n* (%)	157 (57.9)
Surgery, *n* (%)	114 (42.1)
Elective operation, *n* (%)	17 (6.3)
Emergency operation, *n* (%)	97 (35.8)
*ICU admission category*^b^	
Cardiovascular, *n* (%)	34 (12.6)
Respiratory, *n* (%)	88 (32.7)
Gastrointestinal, *n* (%)	56 (21.9)
Trauma, *n* (%)	11 (4.1)
Other, *n* (%)	79 (29.3)
*Sepsis at ICU admission*	
Sepsis, *n* (%)	146 (53.7)
Septic shock, *n* (%)	136 (50.0)
APACHE II score at ICU admission–median [IQR]	23 [17–29]
SOFA score at ICU admission–median [IQR]	8 [6–10]
Endotracheal intubation duration–median [IQR], days	5.3 [3.3–7.5]
*Organ support use*	
Vasopressor, *n* (%)	212 (77.9)
ECMO, *n* (%)	6 (2.2)
IABP, *n* (%)	13 (4.8)
RRT, *n* (%)	83 (30.5)
Enteral nutrition, *n* (%)	219 (80.5)
Enteral nutrition duration–median [IQR], days	10 [5–19]
Parenteral nutrition, *n* (%)	73 (26.8)
Parenteral nutrition duration–median [IQR], days	8 [4–15]
SOFA score on extubation day–median [IQR]	4 [2–6]
Delirium on extubation day, *n* (%)	139 (51.1)
Length of ICU stay–median [IQR], days	8 [5–11]
Length of hospital stay–median [IQR], days	44 [27–67]

IQR: Interquartile range, BMI: body mass index, ICU: intensive care unit, APACHE: acute physiologic and chronic health evaluation, SOFA: Sequential Organ Failure Assessment, ECMO: extracorporeal membrane oxygenation, IABP: intra-aortic balloon pump, RRT: renal replacement therapy

^a^Of 272 patients, one was missing. ^b^Of 272 patients, three were missing

**Table 2:** Swallow screening test and characteristics of SLT

Variables	All (*n* = 272)
*Swallow screening*	
MWST score–median [IQR]	3 (2–3)
*Speech and language therapy*	
Time interval from extubation to SLT initiation–median [IQR], days	1.0 (0.3–2.2)
Time interval from extubation to oral intake–median [IQR], days^a^	4 (2–9)
Frequency of SLT–median [IQR], days per week	4 (3–5)
Swallowing rehabilitation, *n* (%)	259 (95.2)
Compensatory rehabilitation, *n* (%)	247 (90.8)

SLT: Speech and language therapy, MWST: modified water swallowing test, IQR: interquartile range

^a^Of 272 patients, 29 were unable to resume oral intake during hospitalization

**Table 3:** Multivariable logistic regression analysis of association between the timing of SLT initiation and outcomes

Outcomes	All (*n* = 272)	Unadjusted OR (95% CI)	*p*-value	Adjusted OR (95% CI)	*p*-value
*Primary outcomes*					
Dysphagia or death on hospital discharge, n (%)	82 (30.1)	1.12 (1.04–1.20)	0.002	1.09 (1.02–1.18)	0.009
*Secondary outcomes*					
Dysphagia or death at the 7th day after extubation, *n* (%)	188 (69.1)	1.36 (1.13–1.65)	0.001	1.28 (1.05–1.55)	0.011
Dysphagia or death at the 14th day after extubation, *n* (%)^a^	137 (51.8)	1.38 (1.18–1.61)	< 0.001	1.34 (1.13–1.58)	< 0.001
Dysphagia or death at the 28th day after extubation, *n* (%)^b^	92 (42.9)	1.21 (1.08–1.36)	0.001	1.21 (1.07–1.36)	0.001
Aspiration pneumonia after extubation, *n* (%)	79 (29.0)	1.11 (1.04–1.19)	0.002	1.09 (1.02–1.17)	0.012
In-hospital mortality, *n* (%)	31 (11.4)	1.04 (0.98–1.11)	0.135	1.04 (0.97–1.12)	0.203

Variables for the outcomes in the multivariable logistic regression included timing of SLT initiation, institutions, age, ICU admission type, preexisting dementia, cerebrovascular disease, duration of mechanical ventilation, delirium on the day of extubation, SOFA score on the day of extubation, EN, and PN. SLT: speech and language therapy, CI: confidence interval, OR: odds ratio, ICU: intensive care unit, SOFA: Sequential Organ Failure Assessment, EN: enteral nutrition, PN: parenteral nutrition

^a^Of 272 patients, eight were missing

^b^Of 272 patients, 58 were missing

In the sensitivity analysis, our results were unaffected by alternating the definition of dysphagia (Additional file 2), excluding the patients who had gastrointestinal surgery (Additional file 3), excluding those who died after SLT (Additional file 4), or developing other models using different covariates (Additional file 5). Similarly, those who started SLT within one day after extubation were associated with a decrease in dysphagia or death at hospital discharge and aspiration pneumonia (Additional file 6).

## Discussion

In this multicenter retrospective cohort study conducted on data from patients treated in the ICUs of eight hospitals in Japan, we evaluated the timing of SLT initiation after extubation and dysphagia or death. We found that more than half of the patients with PED were provided SLT within 24 h after extubation. A delay in initiation of SLT post-extubation was associated with dysphagia or death at hospital discharge. Our findings suggest that initiation of SLT within 24 h after extubation in patients with swallowing disorders might be preferable in terms of preventing this important complication in the ICU.

While “disability-free survival” can be used as a valid patient-centered endpoint in perioperative outcomes research, it can be equally useful for shared decision making, quality metrics, and benchmarking quality of care. Disability-free survival is a combination of survival (1—mortality) and a patient-reported assessment of disability measured with a validated questionnaire.

We used dysphagia or death at hospital discharge as a primary outcome. Although it can complicate the interpretation, using this composite outcome offers several advantages. First, this outcome allows us to evaluate disability-free survival, which is considered a valid patient-centered outcome as a composite outcome of mortality and morbidity [26]. As a matter of fact, recent studies have evaluated disability-free survival as a composite outcome, integrating all-cause mortality, regardless of the study design [20, 21, 27, 28]. Second, it can achieve sufficient power to detect a treatment effect. A composite outcome has been proposed to enhance statistical efficiency for critical care trials [29]. Third, this composite outcome can reduce selection bias. Excluding patients who died after initiation of SLT prevents us from examining the swallowing function and the effect of SLT, thus resulting in selection bias. The primary outcome in this study was selected based upon the rationale that the composite of dysphagia or death could minimize selection bias by accounting for death as a competing risk. Nonetheless, similar results were obtained even after excluding those patients who died after SLT initiation.

The underlying pathophysiological mechanism of PED is multifactorial, including direct trauma, neuromyopathy as part of ICU-acquired weakness, impaired oropharyngeal and laryngeal sensation, altered level of consciousness, gastroesophageal reflux, and discoordination of breathing and swallowing [10, 24, 30]. Given these proposed mechanisms, early intervention against PED is a potential efficient strategy, similar to how early mobilization appears to prevent the development of ICU-acquired weakness [31]. Although several studies have shown a favorable efficacy of swallowing and oral care or speech therapy delivered by specially trained nurses or SLPs on PED [25, 32], optimal timing for the initiation of SLT has not been explored. Several studies have looked at the timing of dysphagia assessment or SLT among non-PED patients. In a large prospective cohort study, delays in dysphagia screening were associated with a greater risk of pneumonia in acute stroke patients [33]. It was speculated that this phenomenon resulted from inappropriate initiation of oral intake or delayed care for ongoing dysphagia. Moreover, a previous study reported that early oral care and SLT (within 24 h after admission) improved swallowing function and survival after hemorrhagic stroke [34]. Another investigation also showed similar results, where an early intervention group (three days after stroke) had better swallowing performance compared a late post-stroke intervention group [35]. Early initiation of SLT, as shown in our study, may be a beneficial strategy to improve swallowing dysfunction and prevent subsequent adverse events post-extubation in critically ill patients.

Although detailed time metrics were not available, SLT was provided later than 24 h after extubation during the ICU stay in past studies [8, 25]. In these studies, more than half of the patients with dysphagia received their first SLT session within 24 h following extubation. It might be challenging to initiate SLT within this timeframe in terms of clinical feasibility and given the expectation of recovery from dysphagia within 24–48 h post-extubation [36–38]. Nonetheless, this strategy of early SLT initiation might have contributed to early resolution of dysphagia; our results showed that swallowing function had not been restored in 30% of PED patients at hospital discharge, which was less than a previous prospective observational study (58/90, 64%), despite a higher patient age and severity in our study subjects [4].

To our knowledge, this study was the first to link the timing of SLT in PED patients in the ICU with their outcomes. According to the results from national surveys, many ICUs have become aware of this important issue; however, standardized therapeutic strategies to treat PED have not yet been established [2, 39, 40]. Furthermore, a recent large international survey revealed that SLPs were not yet even available in approximately one third of ICUs [41], which implies that outcome effects could be different from the findings of this study in the setting of absence of SLPs in the ICU. Although further research is warranted to determine and validate proper timing of SLT and which patients to prioritize, implementing early initiation of SLT could be a part of a global strategy to reduce the incidence of persistent PED.

## Limitations

First, the timing of SLT initiation was completely dependent on the physician’s preference. Why SLT was delayed for some patients was uncertain: It might have been attributed to patient factors and/or physicians expecting spontaneous recovery. To minimize the bias, in addition to institutions, SOFA scores and the presence or absence of delirium on the day of extubation, which might lead to a delay in starting SLT, were taken into consideration in the multivariable analysis. Due to retrospective design of the study, we still must acknowledge that uncaptured clinical data such as trajectories of actual respiratory or mental status post-extubation may play a causative role in SLT initiation timing. Second, this study did not collect data on the size of the endotracheal tube. A recent prospective study found that larger endotracheal tube size induced post-extubation aspiration in patients with acute respiratory failure [35]. Related to the first limitation, additional unmeasured or potential cofounders may exist. To overcome these limitations, a randomized controlled trial should be conducted. Third, all participants were screened for PED using the MWST; however, instrumental assessment via FEES or VFSS has become implemented in recent years because these provide comprehensive picture of the swallowing process [42]. A previous prospective study showed that the FEES was safe, well tolerated, and significantly correlated with FOIS score [42]. In terms of feasibility, FEES, which can be performed at the bedside, would be preferable to VFSS in the ICU setting [43]. Nonetheless, the MWST was deemed a pragmatic bedside assessment without requiring additional equipment or resources [14, 15]. Forth, daily SLT was completely dependent on the local policies within the above-mentioned framework, and practice compliance was not examined. Therefore, standardized and validated approach to care PED patients should be established to ensure our findings. Finally, long-term follow-up may be needed, considering the profound impact of PED on quality of life.

## Conclusions

In this retrospective analysis, we found that delayed timing of SLT initiation was associated with poor outcomes such as persistent dysphagia or death and aspiration pneumonia after extubation. Our observations indicate that the initiation of SLT within 24 h after extubation in patients with PED appears to be a favorable strategy for early recovery from swallowing disorders in the ICU. Since this was a retrospective study, a randomized controlled trial seems warranted to confirm these results.

## Supplementary Information

**Additional file 1**: Characteristics of each institution and ICU

**Additional file 2**: Multivariable logistic regression analysis of association between the timing of SLT initiation and outcomes, alternating the definition of dysphagia as FOIS < 6

**Additional file 3**: Multivariable logistic regression analysis of association between the timing of SLT initiation and outcomes, excluding those who had gastrointestinal surgery

**Additional file 4**: Multivariable logistic regression analysis of association between the timing of SLT initiation and outcomes, excluding fatal cases

**Additional file 5**: Multivariable logistic regression analysis of association between the timing of SLT initiation and outcomes, adjusting for different covariates

**Additional file 6**: Multivariable logistic regression analysis of association between the timing of SLT initiation and outcomes, stratifying the population into three groups.

## Data Availability

The datasets from this study are available from the corresponding author upon request.

## Abbreviations

AOR: Adjusted odds ratio
APACHE: Acute physiologic and chronic health evaluation
BMI: Body mass index
CI: Confidence interval
ECMO: Extracorporeal membrane oxygenation
EN: Enteral nutrition
FEES: Flexible endoscopic evaluation of swallowing
FOIS: Functional oral intake scale
IABP: Intra-aortic balloon pump
ICU: Intensive care unit
IQR: Interquartile range
MWST: Modified water swallowing test
OR: Odds ratio
PED: Post-extubation dysphagia
PN: Parenteral nutrition
RRT: Renal replacement therapy
SLT: Speech and language therapy
SLP: Speech and language pathologist
SOFA: Sequential Organ Failure Assessment
VFSS: Videofluoroscopic swallowing study
