# Supplementary material for: Association between timing of speech and language therapy initiation and outcomes among post-extubation dysphagia patients: a multicenter retrospective cohort study
Source: Crit Care. 2022 Apr 8;26:98. doi: 10.1186/s13054-022-03974-6 (PMC8991938; doi:10.1186/s13054-022-03974-6)
Supplement: Supplementary file 1 — Additional file 1: Characteristics of each institution and ICU [file 13054_2022_3974_MOESM1_ESM.docx]

**Additional File 1.** Characteristics of each institution and ICU.

Institutions 1^a^ 2^b^ 3^c^ 4^d^ 5^e^ 6^f^ 7^g^ 8^h^

No. of ICU beds, n 10 10 10 30 10 6 4 14

No. of intensivists, n 2 5 0 2 0 3 4 7

No. of SLPs in the hospital, n 3 4 3 6 3 16 4 6

No. of dedicated SLPs in the ICU, n 0 0 0 0 0 1 0 0

No. of nurses in the ICU, n 32 36 34 86 20 40 16 43

No. of ICU admissions per year, n 923 912 240 2400 453 1575 114 1185

Type of ICU

　Mixed medical-surgical ICU Yes Yes Yes Yes Yes Yes Yes Yes

Types of ICU models

Closed ICU model No Yes No No No No No Yes

Open ICU model No No Yes No Yes No No No

Intensivist co-management model Yes No No Yes No Yes Yes No

ICU: intensive care unit, SLP: speech and language pathologist

a: Okayama Saiseikai General Hospital, b: Nara Prefecture General Medical Center, c: Okayama Rosai Hospital, d: Seirei Hamamatsu General Hospital, e: Misato Kenwa Hospital, f: Nagoya Medical Center, g: Hokkaido Medical Center, h: Kameda Medical Center
