# Supplementary material for: Association between timing of speech and language therapy initiation and outcomes among post-extubation dysphagia patients: a multicenter retrospective cohort study
Source: Crit Care. 2022 Apr 8;26:98. doi: 10.1186/s13054-022-03974-6 (PMC8991938; doi:10.1186/s13054-022-03974-6)
Supplement: Supplementary file 2 — Additional file 2: Multivariable logistic regression analysis of association between the timing of SLT initiation and outcomes, alternating the definition of dysphagia as FOIS < 6 [file 13054_2022_3974_MOESM2_ESM.docx]

**Additional File 2.** Multivariable logistic regression analysis of association between the timing of SLT initiation and outcomes, alternating the definition of dysphagia as FOIS<6.

Outcomes, No. (%) All (n=273) Unadjusted OR (95% CI) p-value Adjusted OR (95% CI) p-value

**Primary Outcomes**

Dysphagia or death at hospital discharge 123 (45.2) 1.12 (1.04-1.20) 0.002 1.11 (1.01-1.21) 0.024

**Secondary Outcomes**

Dysphagia or death on the 7th day after extubation 237 (87.1) 2.21 (1.37-3.55) 0.001 1.99 (1.25-3.17) 0.003

Dysphagia or death on the 14th day after extubation ^a^ 197 (74.6) 1.62 (1.23-2.14) 0.001 1.48 (1.10-1.98) 0.008

Dysphagia or death on the 28th day after extubation ^b^ 137 (64.0) 1.29 (1.09-1.54) 0.003 1.24 (1.04-1.48) 0.015

Variables for the outcomes in the multivariable logistic regression included timing of SLT initiation, institutions, age, ICU admission type, pre-existing dementia, cerebrovascular disease, duration of mechanical ventilation, delirium on the day of extubation, SOFA score on the day of extubation, EN, and PN. SLT: speech and language therapy, FOIS: function oral intake scale, CI: confidence interval, OR: odds ratio, ICU: intensive care unit, SOFA: sequential organ failure assessment, EN: enteral nutrition, PN: parenteral nutrition

^a^ Of 272 patients, eight were missing.

^b^ Of 272 patients, 58 were missing.
