# Supplementary material for: Association between timing of speech and language therapy initiation and outcomes among post-extubation dysphagia patients: a multicenter retrospective cohort study
Source: Crit Care. 2022 Apr 8;26:98. doi: 10.1186/s13054-022-03974-6 (PMC8991938; doi:10.1186/s13054-022-03974-6)
Supplement: Supplementary file 3 — Additional file 3: Multivariable logistic regression analysis of association between the timing of SLT initiation and outcomes, excluding those who had gastrointestinal surgery [file 13054_2022_3974_MOESM3_ESM.docx]

**Additional File 3.** Multivariable logistic regression analysis of association between the timing of SLT initiation and outcomes, excluding those who had gastrointestinal surgery.

Outcomes, No. (%) All (n=224) Unadjusted OR (95% CI) p-value Adjusted OR (95% CI) p-value

**Primary Outcomes**

Dysphagia or death at hospital discharge 72 (32.1) 1.13 (1.03-1.23) 0.005 1.11 (1.02-1.22) 0.016

**Secondary Outcomes**

Dysphagia or death on the 7th day after extubation 156 (69.6) 1.39 (1.12-1.72) 0.003 1.33 (1.07-1.67) 0.010

Dysphagia or death on the 14th day after extubation ^a^ 114 (52.2) 1.33 (1.14-1.56) <0.001 1.33 (1.11-1.58) 0.001

Dysphagia or death on the 28th day after extubation ^b^ 80 (44.6) 1.17 (1.05-1.31) 0.004 1.20 (1.06-1.36) 0.003

Aspiration pneumonia 71 (31.7) 1.14 (1.04-1.24) 0.004 1.11 (1.01-1.22) 0.018

In-hospital mortality 28 (12.5) 1.02 (0.92-1.13) 0.661 1.00 (0.89-1.13) 0.908

Variables for the outcomes in the multivariable logistic regression included timing of SLT initiation, institutions, age, ICU admission type, pre-existing dementia, cerebrovascular disease, duration of mechanical ventilation, delirium on the day of extubation, SOFA score on the day of extubation, EN, and PN. SLT: speech and language therapy, CI: confidence interval, OR: odds ratio, ICU: intensive care unit, SOFA: sequential organ failure assessment, EN: enteral nutrition, PN: parenteral nutrition

^a^ Of 224 patients, six were missing.

^b^ Of 224 patients, 55 were missing.
