# Supplementary material for: Association between timing of speech and language therapy initiation and outcomes among post-extubation dysphagia patients: a multicenter retrospective cohort study
Source: Crit Care. 2022 Apr 8;26:98. doi: 10.1186/s13054-022-03974-6 (PMC8991938; doi:10.1186/s13054-022-03974-6)
Supplement: Supplementary file 4 — Additional file 4: Multivariable logistic regression analysis of association between the timing of SLT initiation and outcomes, excluding fatal cases [file 13054_2022_3974_MOESM4_ESM.docx]

**Additional File 4.** Multivariable logistic regression analysis of association between the timing of SLT initiation and outcomes, excluding fatal cases.

Outcomes, No. (%) All (n=241) Unadjusted OR (95% CI) p-value Adjusted OR (95% CI) p-value

**Primary Outcomes**

Dysphagia at hospital discharge 52 (21.5) 1.13 (1.04-1.23) 0.002 1.10 (1.02-1.20) 0.012

**Secondary Outcomes**

Dysphagia on the 7th day after extubation 161 (66.8) 1.37 (1.13-1.67) 0.001 1.28 (1.05-1.57) 0.014

Dysphagia on the 14th day after extubation ^a^ 111 (47.4) 1.38 (1.18-1.61) <0.001 1.33 (1.12-1.57) 0.001

Dysphagia on the 28th day after extubation ^b^ 71 (37.7) 1.23 (1.09-1.39) 0.001 1.20 (1.06-1.36) 0.003

Aspiration pneumonia 60 (24.9) 1.18 (1.07-1.29) <0.001 1.14 (1.04-1.26) 0.004

Variables for the outcomes in the multivariable logistic regression included timing of SLT initiation, institutions, age, ICU admission type, pre-existing dementia, cerebrovascular disease, duration of mechanical ventilation, delirium on the day of extubation, SOFA score on the day of extubation, EN, and PN. SLT: speech and language therapy, CI: confidence interval, OR: odds ratio, ICU: intensive care unit, SOFA: sequential organ failure assessment, EN: enteral nutrition, PN: parenteral nutrition.

^a^ Of 241 patients, six were missing.

^b^ Of 241 patients, 53 were missing.
