# Supplementary material for: Association between timing of speech and language therapy initiation and outcomes among post-extubation dysphagia patients: a multicenter retrospective cohort study
Source: Crit Care. 2022 Apr 8;26:98. doi: 10.1186/s13054-022-03974-6 (PMC8991938; doi:10.1186/s13054-022-03974-6)
Supplement: Supplementary file 5 — Additional file 5: Multivariable logistic regression analysis of association between the timing of SLT initiation and outcomes, adjusting for different covariates [file 13054_2022_3974_MOESM5_ESM.docx]

**Additional File 5.** Multivariable logistic regression analysis of association between the timing of SLT initiation and outcomes, adjusting for different covariates.

Outcomes Model 2 Model 3

Adjusted OR (95% CI) p-value Adjusted OR (95% CI) p-value

**Primary Outcomes**

Dysphagia or death at hospital discharge 1.08 (1.00-1.15) 0.025 1.07 (1.00-1.15) 0.025

**Secondary Outcomes**

Dysphagia or death on the 7th day after extubation 1.25 (1.03-1.52) 0.020 1.26 (1.04-1.53) 0.019

Dysphagia or death on the 14th day after extubation ^a^ 1.32 (1.12-1.56) 0.001 1.32 (1.12-1.56) 0.001

Dysphagia or death on the 28th day after extubation ^b^ 1.19 (1.06-1.34) 0.003 1.19 (1.06-1.35) 0.003

Aspiration pneumonia after extubation 1.07 (1.00-1.15) 0.046 1.07 (1.00-1.15) 0.047

In-hospital mortality 1.05 (0.97-1.13) 0.170 1.05 (0.97-1.13) 0.171

Model 2: Variables for the outcomes in the multivariable logistic regression included timing of SLT initiation, gender, age, BMI, SOFA score at ICU admission, sepsis, and MWST score. Model 3: Variables for the outcomes in the multivariable logistic regression included timing of SLT initiation, duration of mechanical ventilation, vasopressor, ECMO, IABP, and RRT use, EN, and PN. SLT: speech and language therapy, CI: confidence interval, OR: odds ratio, BMI: body mass index, SOFA: sequential organ failure assessment, ICU: intensive care unit, MWST: modified water-swallowing test, ECMO: extracorporeal membrane oxygenation, IABP: intra-aortic balloon pump, RRT: renal replacement therapy, EN: enteral nutrition, PN: parenteral nutrition,

^a^ Of 272 patients, eight were missing.

^b^ Of 272 patients, 58 were missing.
