# Supplementary material for: Association between timing of speech and language therapy initiation and outcomes among post-extubation dysphagia patients: a multicenter retrospective cohort study
Source: Crit Care. 2022 Apr 8;26:98. doi: 10.1186/s13054-022-03974-6 (PMC8991938; doi:10.1186/s13054-022-03974-6)
Supplement: Supplementary file 6 — Additional file 6: Multivariable logistic regression analysis of association between the timing of SLT initiation and outcomes, stratifying the population into three groups. [file 13054_2022_3974_MOESM6_ESM.docx]

**Additional File 6.** Multivariable logistic regression analysis of association between the timing of SLT initiation and outcomes, stratifying the population into three groups.

Timing of SLT initiation, day ≤1 ≤2 2<

Adjusted OR (95% CI) Adjusted OR (95% CI) Adjusted OR (95% CI)

**Primary Outcomes**

Dysphagia or death at hospital discharge 0.48 (0.26-0.87) 0.74 (0.40-1.39) 1.33 (0.71-2.48)

**Secondary Outcomes**

Dysphagia or death on the 7th day after extubation 0.40 (0.21-0.78) 0.31 (0.13-0.70) 3.20 (1.41-7.26)

Dysphagia or death on the 14th day after extubation ^a^ 0.24 (0.13-0.43) 0.24 (0.12-0.49) 4.02 (2.01-8.03)

Dysphagia or death on the 28th day after extubation ^b^ 0.35 (0.18-0.67) 0.39 (0.19-0.79) 2.51 (1.25-5.06)

Aspiration pneumonia 0.52 (0.28-0.96) 0.53 (0.28-1.01) 1.85 (0.98-3.49)

In-hospital mortality 0.89 (0.39-2.02) 1.23 (0.49-3.11) 0.80 (0.32-2.03)

Variables for the outcomes in the multivariable logistic regression included timing of SLT initiation, institutions, age, ICU admission type, pre-existing dementia, cerebrovascular disease, duration of mechanical ventilation, delirium on the day of extubation, SOFA score on the day of extubation, EN, and PN.

SLT: speech and language therapy, CI: confidence interval, OR: odds ratio, ICU: intensive care unit, SOFA: sequential organ failure assessment, EN: enteral nutrition, PN: parenteral nutrition

^a^ Of 272 patients, eight were missing.

^b^ Of 272 patients, 58 were missing.
